# 1-(2-Furylmethyl­ene)-2-(2-nitro­phen­yl)hydrazine

**DOI:** 10.1107/S1600536809037052

**Published:** 2009-09-26

**Authors:** Zhi-Gang Yin, Zong-Lei Fei, Heng-Yu Qian, Xue-Wen Zhu, Chun-Xia Zhang

**Affiliations:** a Key Laboratory of Surface and Interface Science of Henan, School of Materials & Chemical Engineering, Zhengzhou University of Light Industry, Zhengzhou 450002, People’s Republic of China

## Abstract

The title Schiff base compound, C_11_H_9_N_3_O_3_, was obtained from a condensation reaction of furan-2-carbaldehyde and 2-nitro­phenyl­hydrazine. The mol­ecule is roughly planar, the largest deviation from the mean plane defined by all non-H atoms being 0.097 (4). An in ntra­molecular N—H⋯O hydrogen bond might influence the planar conformation of the mol­ecule. In the crystal, weak C—H⋯O hydrogen bonds link the mol­ecules, forming a chain.

## Related literature

For the role played by Schiff base compounds in the development of various proteins and enzymes, see: Kahwa *et al.* (1986 ▶); Santos *et al.* (2001 ▶).
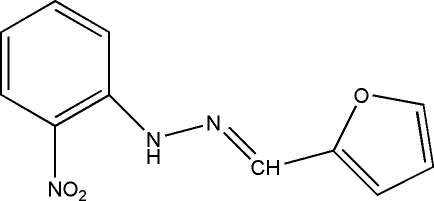


## Experimental

### 

#### Crystal data

C_11_H_9_N_3_O_3_
*M* *_r_* = 231.21
Monoclinic, 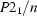

*a* = 15.852 (3) Å
*b* = 3.8000 (12) Å
*c* = 17.721 (4) Å
β = 97.89 (2)°
*V* = 1057.4 (5) Å^3^
*Z* = 4
Mo *K*α radiation
μ = 0.11 mm^−1^
*T* = 296 K
0.21 × 0.19 × 0.17 mm

#### Data collection

Bruker SMART CCD area-detector diffractometer
Absorption correction: multi-scan (*SADABS*; Bruker, 1998 ▶) *T*_min_ = 0.979, *T*_max_ = 0.982
3497 measured reflections
2033 independent reflections
619 reflections with *I* > 2σ(*I*)
*R*_int_ = 0.063

#### Refinement

*R*[*F* ^2^ > 2σ(*F* ^2^)] = 0.070
*wR*(*F* ^2^) = 0.195
*S* = 0.73
2033 reflections
154 parameters
H-atom parameters constrained
Δρ_max_ = 0.24 e Å^−3^
Δρ_min_ = −0.23 e Å^−3^

### 

Data collection: *SMART* (Bruker, 1998 ▶); cell refinement: *SAINT* (Bruker, 1998 ▶); data reduction: *SAINT*; program(s) used to solve structure: *SHELXS97* (Sheldrick, 2008 ▶); program(s) used to refine structure: *SHELXL97* (Sheldrick, 2008 ▶); molecular graphics: *ORTEPIII* (Burnett & Johnson, 1996 ▶), *ORTEP-3 for Windows* (Farrugia, 1997 ▶) and *PLATON* (Spek, 2009 ▶); software used to prepare material for publication: *SHELXL97*.

## Supplementary Material

Crystal structure: contains datablocks global, I. DOI: 10.1107/S1600536809037052/dn2486sup1.cif

Structure factors: contains datablocks I. DOI: 10.1107/S1600536809037052/dn2486Isup2.hkl

Additional supplementary materials:  crystallographic information; 3D view; checkCIF report

## Figures and Tables

**Table 1:** Hydrogen-bond geometry (Å, °)

*D*—H⋯*A*	*D*—H	H⋯*A*	*D*⋯*A*	*D*—H⋯*A*
N2—H2⋯O1	0.86	1.98	2.599 (5)	128
C2—H2*A*⋯O2^i^	0.93	2.48	3.360 (7)	158

Symmetry code: (i) 
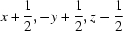
.

**Fig. 1.**
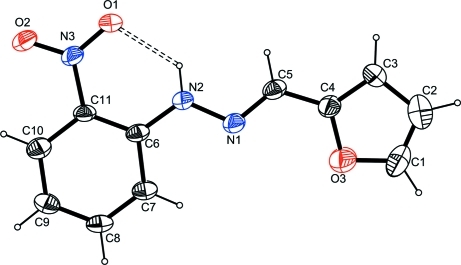
Molecular view of (I) with the atom labeling scheme. Displacement ellipsoids are drawn at the 30% probability level. Hydrogen are represented as small sphere of arbitrary radii. Intramolecular N-H···O hydrogen bond is shown as dashed lines.

**Fig. 2.**
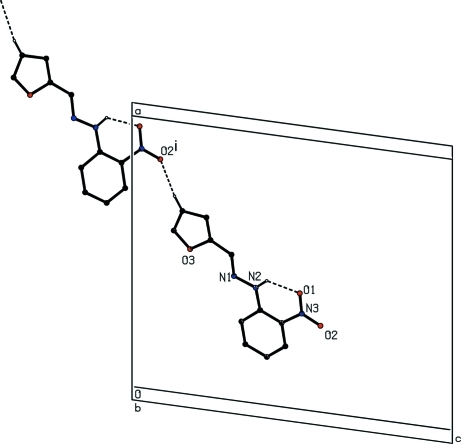
Partial packing view showing the chain formed by C-H···O hydrogen bonds.H atoms not involved in hydrogen bonding have been omitted for clarity. [Symmetry codes: (i) x+1/2, -y+1/2, z-1/2]

## supplementary crystallographic information

### Comment

The chemistry of Schiff base has attracted a great deal of interest in recent years. These compounds play an important role in the development of various proteins and enzymes (Kahwa *et al.*, 1986; Santos *et al.*, 2001). As part of our interest in the study of the coordination chemistry of Schiff bases, we synthesized the title compound and determined its crystal structure.

The whole molecule is roughly planar with the largest deviations from the mean plane being -0.0973 (0.0041) at O3 (Fig. 1). The phenyl and the furan rings are slightly twisted from each other making a dihedral angle of 4.8 (3)°.

The intramolecular N—H···O hydrogen bond might influence the planar conformation of the molecule. Weak intermolecular C-H···O hydrogen bonds link the molecule forming a chain parallel to the (1 0 1) plane (Table 1, Fig. 2).

### Experimental

2-Nitrophenylhydrazine (1 mmol, 0.153 g) was dissolved in anhydrous ethanol (15 ml), The mixture was stirred for several minitutes at 351k, furan-2-carbaldehyde (1 mmol, 0.096 g) in ethanol (8 mm l) was added dropwise and the mixture was stirred at refluxing temperature for 2 h. The product was isolated and recrystallized from methanol, red single crystals of (I) was obtained after 3 d.

### Refinement

All H atoms were positioned geometrically and refined as riding with C—H=0.93 (aromatic), N—H=0.86 Å, and *U*_iso_(H)=1.2*U*_eq_(C).

### Crystal data

**Table:**

C_11_H_9_N_3_O_3_	*F*(000) = 480
*M**_r_* = 231.21	*D*_x_ = 1.452 Mg m^−^^3^
Monoclinic, *P*2_1_/*n*	Mo *K*α radiation, λ = 0.71073 Å
Hall symbol: -P 2yn	Cell parameters from 1960 reflections
*a* = 15.852 (3) Å	θ = 3.2–28.2°
*b* = 3.8000 (12) Å	µ = 0.11 mm^−^^1^
*c* = 17.721 (4) Å	*T* = 296 K
β = 97.89 (2)°	Block, red
*V* = 1057.4 (5) Å^3^	0.21 × 0.19 × 0.17 mm
*Z* = 4	

### Data collection

**Table:**

Bruker SMART CCD area-detector diffractometer	2033 independent reflections
Radiation source: fine-focus sealed tube	619 reflections with *I* > 2σ(*I*)
graphite	*R*_int_ = 0.063
ω scans	θ_max_ = 26.0°, θ_min_ = 3.2°
Absorption correction: multi-scan (*SADABS*; Bruker, 1998)	*h* = −12→19
*T*_min_ = 0.979, *T*_max_ = 0.982	*k* = −4→4
3497 measured reflections	*l* = −21→21

### Refinement

**Table:**

Refinement on *F*^2^	Primary atom site location: structure-invariant direct methods
Least-squares matrix: full	Secondary atom site location: difference Fourier map
*R*[*F*^2^ > 2σ(*F*^2^)] = 0.070	Hydrogen site location: inferred from neighbouring sites
*wR*(*F*^2^) = 0.195	H-atom parameters constrained
*S* = 0.73	*w* = 1/[σ^2^(*F*_o_^2^) + (0.0948*P*)^2^] where *P* = (*F*_o_^2^ + 2*F*_c_^2^)/3
2033 reflections	(Δ/σ)_max_ = 0.006
154 parameters	Δρ_max_ = 0.24 e Å^−^^3^
0 restraints	Δρ_min_ = −0.23 e Å^−^^3^

### Special details

**Table:**

Geometry. All esds (except the esd in the dihedral angle between two l.s. planes) are estimated using the full covariance matrix. The cell esds are taken into account individually in the estimation of esds in distances, angles and torsion angles; correlations between esds in cell parameters are only used when they are defined by crystal symmetry. An approximate (isotropic) treatment of cell esds is used for estimating esds involving l.s. planes.
Refinement. Refinement of *F*^2^ against ALL reflections. The weighted *R*-factor *wR* and goodness of fit *S* are based on *F*^2^, conventional *R*-factors *R* are based on *F*, with *F* set to zero for negative *F*^2^. The threshold expression of *F*^2^ > σ(*F*^2^) is used only for calculating *R*-factors(gt) *etc*. and is not relevant to the choice of reflections for refinement. *R*-factors based on *F*^2^ are statistically about twice as large as those based on *F*, and *R*- factors based on ALL data will be even larger.

### Fractional atomic coordinates and isotropic or equivalent isotropic displacement parameters (Å^2^)

**Table:**

	*x*	*y*	*z*	*U*_iso_*/*U*_eq_	
C1	0.5620 (4)	−0.1566 (18)	0.1304 (3)	0.089 (2)	
H1	0.5467	−0.2470	0.0817	0.107*	
C2	0.6396 (4)	−0.0571 (18)	0.1574 (4)	0.085 (2)	
H2A	0.6878	−0.0666	0.1328	0.103*	
C3	0.6342 (3)	0.0688 (15)	0.2323 (3)	0.0609 (16)	
H3	0.6781	0.1673	0.2659	0.073*	
C4	0.5541 (3)	0.0181 (15)	0.2450 (3)	0.0554 (14)	
C5	0.5169 (3)	0.1022 (14)	0.3106 (2)	0.0508 (14)	
H5	0.5500	0.2188	0.3502	0.061*	
C6	0.3320 (2)	0.0389 (13)	0.3999 (2)	0.0447 (13)	
C7	0.2756 (2)	−0.1246 (13)	0.3415 (3)	0.0521 (14)	
H7	0.2938	−0.1806	0.2952	0.063*	
C8	0.1937 (3)	−0.2002 (14)	0.3537 (3)	0.0560 (14)	
H8	0.1574	−0.3116	0.3154	0.067*	
C9	0.1634 (3)	−0.1163 (15)	0.4210 (3)	0.0591 (15)	
H9	0.1071	−0.1636	0.4269	0.071*	
C10	0.2168 (3)	0.0365 (14)	0.4787 (3)	0.0555 (14)	
H10	0.1979	0.0861	0.5250	0.067*	
C11	0.3009 (2)	0.1184 (13)	0.4672 (2)	0.0446 (12)	
N1	0.4392 (2)	0.0254 (11)	0.3182 (2)	0.0509 (11)	
N2	0.4128 (2)	0.1163 (11)	0.3862 (2)	0.0499 (11)	
H2	0.4473	0.2227	0.4205	0.060*	
N3	0.3518 (2)	0.2903 (12)	0.5299 (2)	0.0535 (12)	
O1	0.42600 (19)	0.3693 (10)	0.52361 (17)	0.0679 (11)	
O2	0.32065 (19)	0.3559 (12)	0.58821 (19)	0.0776 (13)	
O3	0.5066 (2)	−0.1130 (12)	0.1815 (2)	0.0797 (13)	

### Atomic displacement parameters (Å^2^)

**Table:**

	*U*^11^	*U*^22^	*U*^33^	*U*^12^	*U*^13^	*U*^23^
C1	0.111 (5)	0.101 (6)	0.059 (4)	0.020 (5)	0.027 (4)	−0.012 (4)
C2	0.081 (4)	0.086 (5)	0.095 (5)	0.018 (4)	0.035 (4)	0.020 (5)
C3	0.046 (3)	0.072 (4)	0.065 (4)	−0.005 (3)	0.008 (2)	−0.002 (3)
C4	0.048 (3)	0.073 (4)	0.042 (3)	0.014 (3)	−0.003 (2)	−0.003 (3)
C5	0.052 (3)	0.055 (4)	0.042 (3)	0.010 (3)	−0.006 (2)	−0.007 (3)
C6	0.044 (2)	0.047 (3)	0.039 (3)	0.005 (3)	−0.009 (2)	−0.003 (3)
C7	0.051 (3)	0.057 (4)	0.044 (3)	0.000 (3)	−0.011 (2)	−0.001 (3)
C8	0.051 (3)	0.059 (4)	0.051 (3)	−0.004 (3)	−0.016 (2)	−0.008 (3)
C9	0.040 (2)	0.070 (4)	0.064 (3)	−0.002 (3)	−0.006 (2)	−0.001 (3)
C10	0.056 (3)	0.061 (4)	0.045 (3)	0.003 (3)	−0.005 (2)	0.001 (3)
C11	0.040 (2)	0.053 (3)	0.037 (3)	−0.003 (3)	−0.0077 (19)	−0.002 (3)
N1	0.046 (2)	0.063 (3)	0.042 (2)	0.004 (2)	−0.0018 (16)	−0.008 (2)
N2	0.042 (2)	0.067 (3)	0.039 (2)	−0.003 (2)	−0.0005 (16)	−0.007 (2)
N3	0.046 (2)	0.077 (3)	0.035 (2)	0.005 (2)	−0.0035 (18)	−0.007 (2)
O1	0.0486 (18)	0.107 (3)	0.045 (2)	−0.016 (2)	−0.0029 (14)	−0.015 (2)
O2	0.0599 (19)	0.127 (4)	0.043 (2)	−0.003 (2)	−0.0027 (15)	−0.024 (2)
O3	0.072 (2)	0.104 (4)	0.060 (2)	0.006 (2)	−0.0034 (18)	−0.006 (3)

### Geometric parameters (Å, °)

**Table:**

C1—C2	1.313 (8)	C7—C8	1.376 (5)
C1—O3	1.355 (6)	C7—H7	0.9300
C1—H1	0.9300	C8—C9	1.383 (6)
C2—C3	1.426 (7)	C8—H8	0.9300
C2—H2A	0.9300	C9—C10	1.363 (6)
C3—C4	1.334 (6)	C9—H9	0.9300
C3—H3	0.9300	C10—C11	1.412 (5)
C4—O3	1.360 (5)	C10—H10	0.9300
C4—C5	1.409 (5)	C11—N3	1.437 (5)
C5—N1	1.292 (5)	N1—N2	1.373 (4)
C5—H5	0.9300	N2—H2	0.8600
C6—N2	1.367 (5)	N3—O2	1.230 (4)
C6—C11	1.386 (6)	N3—O1	1.234 (4)
C6—C7	1.415 (6)		
			
C2—C1—O3	112.5 (6)	C7—C8—C9	122.3 (4)
C2—C1—H1	123.7	C7—C8—H8	118.8
O3—C1—H1	123.7	C9—C8—H8	118.8
C1—C2—C3	105.2 (5)	C10—C9—C8	119.4 (4)
C1—C2—H2A	127.4	C10—C9—H9	120.3
C3—C2—H2A	127.4	C8—C9—H9	120.3
C4—C3—C2	106.8 (5)	C9—C10—C11	119.2 (5)
C4—C3—H3	126.6	C9—C10—H10	120.4
C2—C3—H3	126.6	C11—C10—H10	120.4
C3—C4—O3	110.2 (4)	C6—C11—C10	122.0 (4)
C3—C4—C5	128.4 (5)	C6—C11—N3	122.5 (4)
O3—C4—C5	121.2 (4)	C10—C11—N3	115.5 (4)
N1—C5—C4	123.3 (4)	C5—N1—N2	116.5 (4)
N1—C5—H5	118.4	C6—N2—N1	120.4 (4)
C4—C5—H5	118.4	C6—N2—H2	119.8
N2—C6—C11	123.9 (4)	N1—N2—H2	119.8
N2—C6—C7	118.6 (4)	O2—N3—O1	121.6 (4)
C11—C6—C7	117.5 (4)	O2—N3—C11	119.6 (4)
C8—C7—C6	119.4 (5)	O1—N3—C11	118.8 (4)
C8—C7—H7	120.3	C1—O3—C4	105.2 (4)
C6—C7—H7	120.3		

### Hydrogen-bond geometry (Å, °)

**Table:**

*D*—H···*A*	*D*—H	H···*A*	*D*···*A*	*D*—H···*A*
N2—H2···O1	0.86	1.98	2.599 (5)	128
C2—H2A···O2^i^	0.93	2.48	3.360 (7)	158

Symmetry codes: (i) *x*+1/2, −*y*+1/2, *z*−1/2.
